# Association between duration of urinary catheterization and post-operative mobilization following elective cesarean section: A retrospective case-control study in Espoo, Finland

**DOI:** 10.18332/ejm/193602

**Published:** 2024-11-07

**Authors:** Hanna Vihervaara, Antti Väänänen, Marja Kaijomaa

**Affiliations:** 1 University of Eastern Finland, Kuopio, Finland; 2 Department of Obstetrics and Gynecology, Helsinki University Hospital, University of Helsinki, Helsinki, Finland

**Keywords:** cesarean section, urinary catheter, mobilization, enhanced recovery, post-operative pain, maternal satisfaction

## Abstract

**INTRODUCTION:**

Cesarean section is the most common surgery performed on women. The enhanced recovery recommendations are early urinary catheter removal and early mobilization, as essential elements of post-operative care. This study aimed to analyze the association between these elements and whether limiting the catheter treatment duration affects the timing of post-operative mobilization.

**METHODS:**

This retrospective case-control study compared the mobilization of healthy elective cesarean patients under different instructions on urinary catheter removal: cases with a preset catheter removal time (8–12 hours) and controls with catheter removal based on midwife considerations. Apart from the preset time of catheter removal, the routine post-operative care was given by the same personnel without any advice on patient mobilization. Data on patient demographics, surgery details, post-operative medication, first upright mobilization, the length of hospital stay, and patient satisfaction were analyzed.

**RESULTS:**

The study comprised 52 cases and one control for each case (N=104). The mean duration of urinary catheterization was 20.15 ± 6.59 and 11.30 ± 4.20 hours in the control and intervention groups, respectively (p<0.001). A linear regression analysis showed a significant association between the catheter removal time and patient mobilization, when adjusted for maternal background parameters (age, BMI, fear of childbirth diagnosis, prior uterine scar), duration and timing of the surgery, bleeding and post-operative analgesic use (R^2^=0.444, p<0.001). No difference was detected in the length of hospital stay, or patient satisfaction.

**CONCLUSIONS:**

Limiting the duration of urinary catheter therapy is associated with shorter time to post-operative mobilization. A prospective randomized trial would provide more detailed information.

## INTRODUCTION

The cesarean section stands out as the most common obstetric surgery performed on women. The number of cesarian sections has steadily increased over past decades and the worldwide incidence is assumed to reach 30%, i.e. 38 million cesarians deliveries, by 2030^1^. In cesarian section, a urinary catheter is used as it is considered to reduce the risk of surgical bladder injury and post-operative urinary retention. It also enables the assessment of urinary output^2^. However, the use of urinary catheter has also been associated with delayed ambulation, prolonged hospital stay, voiding discomfort, and urinary tract infections^3^. Also, the role of early post-operative mobilization is well-established. It is associated with a reduced incidence of complications like thromboembolism, and it improves pulmonary and bowel function, tissue oxygenation, and insulin resistance^4,5^. Also, rapid return of bowel function and decreased hospital stay are associated with early mobilization^6^.

For almost two decades, the ERAS^®^ (Enhanced Recovery After Surgery) -Society has developed and provided evidence-based guidelines for peri-operative care to improve treatment and patient recovery. The guidelines for cesarean section^6-8^ provide a standardized protocol covering the entire treatment continuum from the preoperative preparation to the hospital discharge. Early urinary catheter removal and early mobilization are among the key elements of post-partum ERAS-protocol^9,10^, and the routine placement of catheter has therefore been criticized and more selective usage encouraged^4^. When the catheter is used, immediate or early removal of catheter is advised by the ERAS-guidelines^6^. Also, early post-operative mobilization is strongly recommended by the ERAS^®^-Society^6^.

In Finland, the cesarean-section rate is steadily increasing with operative deliveries accounting for 20.1% of all deliveries in 2023. Meanwhile the rate of planned cesarean sections is still relatively limited, 8.3 %^11^. Most of the cesarean sections are performed from the Pfannenstiel-incision under neuraxial anesthesia utilizing spinal or epidural morphine as part of the multimodal analgesia regimen. The fear for neuraxial morphine-induced urinary retention has been one of the rationales for prolonged use of urinary catheter post operatively^12^. The current institutional practice is to keep the urinary catheters in place until the first post-operative day.

Since avoiding urinary catheter altogether during cesarean section has been associated with markedly shorter duration until ambulation, we hypothesize that actively limiting the catheterization time would be associated with shorter time to ambulation compared to the conventional practice^3^.

The primary aim of the study is to analyze the association between urinary catheterization and mobilization following a planned elective cesarean delivery. The length of post-operative hospital stay, and maternal satisfaction, are assessed as secondary outcomes.

## METHODS

### Study design and setting

This retrospective case-control study is part of a wider research protocol and was conducted at the Espoo Delivery Hospital, affiliated with the Helsinki University Women’s Clinic, in Espoo, Finland. The hospital handles low-risk deliveries and, in 2023, managed 4200 deliveries. The study’s recruitment period was from 28 November 2022 to 4 December 2023, and the follow-up period for each parturient extended from surgery to hospital discharge.

All patients were hospitalized for an elective cesarean section. The intervention group consisted of patients enrolled in an ongoing open-label prospective study evaluating bladder function after early post-operative (8–12 hours) urinary catheter removal. The study protocol focuses solely on bladder function and does not address mobilization. For each case in the intervention group, the next elective cesarean section not enrolled in the study was used as a control. The catheter removal in the control group was based on midwife considerations.

Before surgery, all patients in both groups received uniform written instructions regarding post-operative care, including guidance on early mobilization. The procedures were predominantly performed by junior doctors in the early stages of their careers, always under spinal anesthesia (bupivacaine 10–12 mg, fentanyl 15 µg, morphine 120 µg). Post-operative pain management consisted of ibuprofen 600 mg and paracetamol 1000 mg three times daily, unless contraindicated. In cases of insufficient pain control, additional oral oxycodone (5–10 mg) was administered during the hospital stay. According to the hospital policy, no opioid medications were prescribed for home use.

After the operation, all patients were treated in either of the two postpartum wards and received care from the same healthcare personnel throughout the study period.

### Participants

Based on the historical data, the time to mobilization was expected to be 13 ± 5 hours after the end of the operation. The number of participants needed per group to detect a three-hour reduction in mobilization time in the intervention group was calculated to be 44 (α=0.05, β=0.20). To account for potential missing data, the sample size was increased to 52 participants per group. The study was conducted in our low-risk delivery unit, which restricted the number of eligible parturients to those for whom an elective cesarean section could be performed in this unit. This also formed the inclusion criteria for the study:

- Parturient coming for an elective cesarean delivery in the Espoo Hospital low-risk unit.
- Signed informed consent (in Finnish) from cases in the intervention group. The next available Finnish speaking parturient undergoing an elective cesarean delivery and not participating in the study selected as a control.

Exclusion criteria were based on the admission criteria for the Espoo Hospital:

- Suspicion of abnormal placentation
- Signs of severe preeclampsia
- Known coagulation disorder
- Type I or Type II diabetes
- BMI over 40 kg/m^2^
- Antenatal anticoagulant therapy
- Any comorbidity requiring medical observation or intervention during the peripartum period
- Alcohol or other substance abuse during pregnancy
- Known or suspected difficult airway
- Suspected surgical difficulties (i.e. a maximum of one uncomplicated cesarean section was permitted)

The cases in the intervention group were verified based on the existence of a signed consent to participate in the study, which specified a predetermined time (8, 10, or 12 hours) for the removal of the urinary catheter. These cases were counted as intervention cases regardless of the actual catheter removal time. Data for both the cases and controls were retrieved from the electronic patient data system for both groups.

### Variables

All time points related to the operation and recovery were derived from the hospital’s patient management system (Apotti/Epic Systems Corporation, USA), which requires input on basic parameters, documentation of mobilization data, and catheter removal timestamps.

*Main outcome: time to post-operative mobilization*

Calculated as the time (in hours) from the end of the operation until the parturient is either standing or walking.

*Secondary outcome: length of hospital stay*

Calculated as the time (in hours) from the operation until discharge from the maternity ward. No parturient in this study was transferred to another hospital for subsequent treatment.

*Secondary outcome: maternal satisfaction*

Routinely collected 1–2 days after delivery by midwives as part of the pre-dismissal interview and information session at the maternity ward. Overall satisfaction is measured on a 100 mm visual analog scale, with values expressed from 0 mm (complete dissatisfaction) to 100 mm (very satisfied). If a parturient scored <50 mm on the VAS scale, she was offered a post-dismissal visit to discuss her delivery experience.

*Exposure: time of urinary catheterization*

Time (in hours) from the end of the operation to the removal of the urinary catheter. A target time of 8, 10, or 12 hours was set for intervention cases. These cases were classified as intervention cases regardless of whether the catheter was removed within the target time. The removal time was recorded and verified from nursing notes.

*Potential confounders*

Maternal characteristics (age, weight, BMI, pre-existing fear of childbirth, and prior cesarean delivery) and indications for cesarean section were obtained from the hospital database. Surgery details, such as operation length and blood loss, were documented to identify potential difficulties, and the use of post-operative medications was analyzed to assess pain.

### Bias

All participants in both groups of the study were eligible to have their cesarean section performed in the low-risk delivery unit, thus belonging to the same healthy parturient category. The same standardized anesthesia was used in all cesarean sections. Both groups received the same instructions for mobilization instructions and were cared for by the same staff in the same post-operative department. No stratification by baseline characteristics was performed, and the parturient populations were expected to differ only in terms of participation in the study. The only intervention in the study group was the preset (earlier) removal of the urinary catheter; study participation did not involve additional interventions or monitoring. Data for both groups were collected from the same sources.

### Ethical considerations

The study received approval from the Helsinki University Research Council (Approval No. HUS/730/2022). Based on national legislation (Medical Research Act 488/1999) the need for written informed consent was waived for this retrospective study.

### Statistical analysis

Data analysis was performed using SPSS version 25.0.0 (IBM SPSS Statistics, Armonk, New York). The independent samples t-test and chi-squared test were used to compare continuous and categorical variables, with statistical significance set at p<0.05. Paired t-test was used for the comparison of actual urinary catheterization times with the intended catheterization times. The distribution of descriptive variables (primary indications for cesarean delivery) between groups was compared using the log-rank test, arranged in decreasing order of frequency.

A linear regression model was initially used to assess the association of potential cofactors with the duration of urinary catheterization, irrespective of group assignment. The following adjusted cofactors were included in the analysis: maternal age, BMI, number of prior cesarean sections (zero or one), length of surgery, blood loss during surgery, and the use of oxycodone (mg per 24 hours) during the hospital stay post-operatively. Subsequently, the association of actual urinary catheter use with time to mobilization was tested, along with the same cofactors. The absence of collinearity among cofactors was verified using the variance inflation factor method, with a cut-off value set at below five.

## RESULTS

During the recruitment period for this study, a total of 466 elective cesarean deliveries were performed in the unit. Fifty-two parturients (16% of the Finnish speaking parturients) were successfully recruited into the intervention group, with one control selected for each case. All 52 cases and their respective controls completed the follow-up, with no dropouts. The complete dataset was available for all participants.

There were no statistically significant differences between cases and controls in terms of maternal age (p=0.800), weight (p=0.399), BMI (p=0.097), duration of surgery (p=0.400), blood loss (p=0.545), or diagnosis of fear of childbirth (p=0.556). However, parturients in the control group more frequently had a history of prior cesarean sections (p=0.034) while those in the intervention group had a significantly shorter duration of post-operative urinary catheterization (p<0.001), in line with their assigned study intervention (Table 1).

**Table 1:** Characteristics of the study population and cesarean delivery, Espoo Delivery Hospital, Finland (2023) (N=104)

*Characteristics*	*Intervention group (N=52)*	*Control group (N=52)*	*p^a^*
	*Mean ± SD*	*Mean ± SD*	
**Maternal age** (years)	34.12 ± 4.77	33.87 ± 5.33	0.800
**Maternal weight** (kg)	78.27 ± 12.23	80.55 ± 15.04	0.399
**BMI** (kg/m^2^)	28.55 ± 4.12	30.08 ± 5.14	0.097
**Duration of surgery** (min)	62.58 ± 21.90	66.42 ± 24.41	0.400
**Blood loss** (mL)	654 ± 410	698 ± 309	0.545
	***n (%)***	***n (%)***	
**Prior cesarean section**	11 (21)	21 (40)	0.034
**Fear of childbirth diagnosis**	29 (56)	26 (50)	0.556
**Intended urinary catheter use time** (h), mean ± SD	10.15 ± 1.67		
12	20 (39)		
10	16 (31)		
8	16 (31)		
**Primary indication**			0.369
Fear of childbirth	24 (46)	21 (40)	
Breech presentation	16 (31)	14 (27)	
Prior cesarean delivery	8 (15)	13 (25)	
Excessive fetal growth	1 (1.9)	2 (3.8)	
Pelvic or perineal abnormalities	2 (3.8)	0	
Disproportion	1 (1.9)	1 (1.9)	
Gestational diabetes	0	1 (1.9)	

BMI: body mass index.

a Chi-squared test for categorical variables, t-test for continuous variables, log-rank test for the indications.

The most common indication for cesarean delivery in both groups was maternal fear of childbirth (intervention group 46%, control group 40%), which includes operations performed on maternal request and lacks a specific ICD-10 code. In both groups, the second and third most common indications were breech presentation (intervention group 31%, control group 27%) and prior cesarean delivery (intervention group 15%, control group 25%). A minority of operations were performed due to abnormalities in the maternal vulva, perineum, or pelvis, or due to disproportion, excessive fetal growth, or gestational diabetes. Overall, there were no significant differences in the distribution of primary indications for cesarean delivery between cases in the intervention group and the controls (log-rank test, p=0.369).

### Cofactors associated with the duration of urinary catheterization

Parturients in the study group had a pre-assigned removal time for their urinary catheters, whereas no such time was set for the control group. The actual removal time for urinary catheters in the intervention group was 11.30 ± 4.21 hours, exceeding the target time of 10.15 ± 1.67 hours (paired two-sided t-test, p<0.001). However, the urinary catheterization time in the intervention group was significantly shorter than in the control group, where the mean catheterization time was 20.15 ± 6.59 hours. Post-operative catheterization times are shown in Table 2.

**Table 2:** The duration of urinary catheterization, time to mobilization, parturient satisfaction, and duration of hospital stay, Espoo Delivery Hospital, Finland (2023)

	*Intervention group (N=52) Mean ± SD*	*Control group (N=52) Mean ± SD*	*p^a^*
Duration of urinary catheterization (h)	11.30 ± 4.21	20.15 ± 6.59	<0.001
Time to mobilization (h)^b^	8.86 ± 3.22	12.59 ± 7.00	<0.001
Urinary catheter removed prior to mobilization, n (%)	15 (29)	4 (7.7)	0.005
Patient satisfaction (mm)^c^	90.4 ± 8.40	92.3 ± 9.33	0.352
Duration of post-operative hospitalization (days)	2.49 ± 0.72	2.39 ± 0.73	0.494

a Chi-squared test for the categorical variable, t-test for continuous variables.

b Hours from the end of cesarean section.

c Visual analog scale: 0–100/100 mm.

The association of potential cofactors with the duration of urinary catheterization was assessed using a multiple linear regression model. The overall model was statistically significant [R²=0.429, F(9, 94)=8.174, p<0.001]. The model included maternal age, BMI, prior cesarean section, diagnosis of fear of childbirth, start time of the cesarean section, duration of surgery, blood loss during surgery, post-operative use of oxycodone, and participation in the early catheter removal study as potential cofactors. Among these, only participation in the study significantly predicted the duration of urinary catheterization. The results of the multiple regression analysis are shown in Table 3. There was no significant collinearity among the tested cofactors.

**Table 3:** Cofactor values for the duration of urinary catheterization after the cesarean section and linear regression analysis for their association with the duration of urinary catheterization, Espoo Delivery Hospital, Finland (2023)

	*Mean ± SD*	*B (95% CI)^a^*	*p*	*VIF*
Maternal age (years)	33.99 ± 5.04	-0.076 (-0.315–0.163)	0.528	1.232
BMI (kg/m^2^)	29.31 ± 4.70	-0.017 (-0.265–0.230)	0.891	1.147
Prior cesarean section, n	32	1.678 (-0.954–4.309)	0.209	1.266
Fear of childbirth diagnosis, n	55	-1.205 (-3.436–1.025)	0.286	1.064
Beginning time of operation^b^	11:19 ± 2:06	-0.091 (-0.645–0.462)	0.743	1.153
Duration of surgery (min)	64.50 ± 23.16	-0.046 (-0.099–0.008)	0.095	1.306
Blood loss (mL)	676 ± 362	0.02 (-0.001–0.005)	0.237	1.189
Oxycodone use^c^	2.31 ± 3.21	0.183 (-0.168–0.534)	0.302	1.080
Participation in the study, n	52	-8.408 (-10.66 – -6.15)	<0.001	1.092
Constant		25.37 (10.61–40.13)	<0.001	
R2		0.439		

BMI: body mass index. VIF: variance inflation factor.

a Adjusted unstandardized coefficients.

b Time (hours past midnight).

c Oxycodone p.o. (mg/24-h hospital stay post-operatively).

### Time to mobilization and its association with urinary catheter removal (primary outcome)

The mean time to mobilization for the entire study population was 10.73 ± 5.74 hours. Participation in the early catheter removal study was associated with significantly shorter times to both urinary catheter removal and mobilization post-operatively (Table 2). Urinary catheters were removed prior to mobilization more frequently in the intervention group compared to controls (OR=4.86; 95% CI: 1.49–15.89).

**Figure 1:**
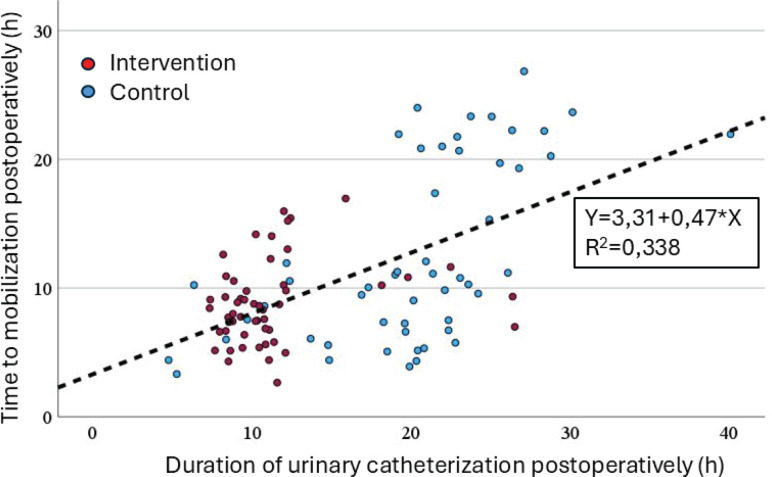
Scatter plot of time to mobilization by duration of urinary catheterization in Espoo Hospital, Finland (N=104) (both parameters counted as hours after end of surgery)

Potential cofactors for post-operative mobilization were examined using a multiple linear regression model, which was statistically significant [R²=0.444, F(10, 93)=7.425, p<0.001]. The model included maternal age, BMI, prior cesarean section, diagnosis of fear of childbirth, start time of the cesarean section, duration of surgery, blood loss during surgery, post-operative use of oxycodone, participation in the early catheter removal study, and actual postoperative catheter use time. Among these, a diagnosis of fear of childbirth was associated with a shorter time to mobilization (p=0.025), while longer urinary catheterization time was associated with a longer time to mobilization (p<0.001). Group assignment (intervention or control) was not associated with the duration until mobilization (p=0.350). There was no significant collinearity among the cofactors. A summary of the linear regression is shown in Table 4. The association between post-operative time to mobilization and urinary catheterization time is depicted in Figure 1.

**Table 4:** Cofactor values for the time to mobilization after the cesarean section and linear regression analysis for their association, Espoo Delivery Hospital, Finland (2023)

	*Mean ± SD*	*B (95% CI)^a^*	*p*	*VIF*
Maternal age (years)	33.99 ± 5.04	0.115 (-0.079–0.310)	0.242	1.237
BMI (kg/m^2^)	29.31 ± 4.70	0.201 (0.000–0.401)	0.050	1.148
Prior cesarean section, n	32	1.019 (-1.136–3.179)	0.350	1.288
Fear of childbirth diagnosis, n	55	-2.095 (-3.917 – -0.273)	0.025	1.077
Beginning time of operation^b^	11:19 ± 2:06	-0.172 (-0.622–0.277)	0.448	1.154
Duration of surgery (min)	64.50 ± 23.16	-0.09 (-0.053–0.0350)	0.690	1.346
Blood loss (mL)	676 ± 362	-0.03 (-0.005–0.000)	0.049	1.207
Oxycodone use^c^	2.31 ± 3.21	0.177 (-0.109–0.464)	0.222	1.092
Duration of urinary catheterization^d^	15.72 ± 7.07	0.477 (0.0310–0.643)	<0.001	1.783
Participation in the study, n	52	1.089 (-1.215–3.393)	0.350	1.728
Constant		-2.387 (15.089–10.315)	0.710	
R^2^		0.444		

BMI: body mass index. VIF: variance inflation factor.

a Adjusted unstandardized coefficients.

b Time (hours past midnight).

c Oxycodone p.o. (mg/24-h hospital stay post-operatively).

d Hours after the end of operation.

## DISCUSSION

To our knowledge, this is the first study in Finland demonstrating that in healthy parturients, the preset limitation of post-cesarean urinary catheterization is associated with faster post-operative mobilization. These two elements were interrelated and limiting the time of catheterization resulted in a positive impact on post-operative mobilization. Our regression analysis revealed that the timing of post-operative mobilization was primarily dependent on the duration of catheterization, rather than participation in the study or other cofactors. The participation in the study was the primary cofactor influencing the duration of catheterization but did not explain the faster post-operative mobilization.

The most common indication for the cesarean section in the cohort was maternal fear of childbirth including surgeries done on maternal request. Interestingly, it had a significant negative association with the duration of catheterization and mobilization. This may be associated with the fact that these parturients most often are young and healthy. The incidence of primary indications did not show significant differences between the groups.

Most elective cesarean sections were completed between 10 a.m. and 3 p.m. and the current standard treatment protocol allows for the removal of the urinary catheter at a time considered convenient. In this study only a limited number (35%) of catheters in the control group were removed before 8 a.m. the following morning, compared to 96% in the intervention group. This may suggest reluctance to remove the catheters at night on the part of either the parturients or staff. Also, previous studies have reported that lack of personnel and limitations in resources can create obstacles in the way of optimal treatment. When the staff is limited, the prolonged use of catheter and the decreased need for patient toileting may be more convenient to the personnel^13,14^. This reality also applies to the assistance in the mobilization of the patient^15,16^. Also, the lack of knowledge, and education are described to be among the most important barriers to achieve optimal catheter use and early mobilization. The studies by Jain et al.^17^ and Niedeshauser et al.^18^ described that the knowledge and perceptions of doctors and nurses regarding the appropriate use of urinary catheter can be variable and inefficient. As far as it concerns the use of catheter in cesarean section, this is understandable, as available information is contradictory. Earlier studies have reported that the lack of education and awareness can also hinder patient’s early mobilization^19^, as well as a working culture that does not prioritize physical activity after surgery^20^.

Most of the patients in this study were mobilized before the removal of the catheter. Our data indicate that mobilization is almost always required for catheter removal, as catheters are generally not removed before the patient is either able or willing to mobilize herself. In the study, a preset target time for catheter removal seemed to motivate both the patient and the personnel towards mobilization, because the mobilization was done significantly later if the catheter removal time was not preset.

Compared to the controls, a lower percentage of parturients with a prior uterine scar was observed in the intervention group. This may be associated with their previous experience of recovering from a cesarean section and a lower willingness to participate in a study involving earlier catheter removal. Previous studies have reported that multiparas undergoing a repeat cesarean delivery may have a significantly higher risk of inadequate post-operative pain management compared to those undergoing their first cesarean^21,22^.

In general, the length of catheter therapy after surgery should be based on justified clinical need. Besides its negative effect on mobilization, inappropriate catheter use increases the risk of urinary infections^23,24^ and causes discomfort to the patient^25,26^. Immediate post-operative removal is recommended by the ERAS guidelines^6^, but concerns regarding impaired bladder function after neuraxial anesthesia and long-acting opioids have been raised^12^. In the absence of detailed data, a recent meta-analysis by Hou et al.^27^ considered the optimal time for removal to be 6 hours after surgery.

Studies concerning the catheter use and patient perspective have conflicting results. The study by Safdar et al.^23^ reported that 45% of the patients considered indwelling urinary catheters convenient because they did not have to get up and go to the bathroom while the study by Liebermann et al.^28^ concluded that the most common barrier to post-operative mobilization was the urinary catheter

As thromboembolic events remain one of the main causes of maternal deaths, early mobilization has a critical prophylactic role in reducing the risk^29^. Compared to non-pregnant, non-postpartum women, the risk of thromboembolic events during the post-partum period is described to be up to 84 times higher^30,31^ and compared to vaginal delivery, the risk after cesarean is four-fold^32^. Even though the absolute risk (3/1000) can be considered low, the role of mobilization cannot be underestimated^32^.

A significant proportion of patients experience high-intensity post-operative pain and require opioids after surgery. A study by Idawati et al.^33^ reported that early mobilization significantly reduced pain levels after cesarean. The present study did not show association between post-operative mobilization and oxycodone use after surgery.

Our study did not show any association between parturient satisfaction and the duration of catheterization, but this may be due to the overall satisfaction index being high in both groups. However, it is notable that shorter urinary catheterization was not associated with lower parturient satisfaction. The study by Ulfa et al.^34^ reported that limitations in mobility and wound pain are factors that interfere with the ability to breastfeed after cesarean. A French study by Laronche et al.^35^ examined post-operative maternal satisfaction and maternal neonatal bonding by comparing hospitals using either ERAS-protocol (i.e. catheter removal ≤12 hours, mobilization, at least sitting, in 6–8 hours) to a more conventional protocol (i.e. urinary catheter removal at 24 hours). They concluded that mothers undergoing the ERAS- protocol treatment were more satisfied with the mother- and baby-relationship and they were mobilized more rapidly. Our study did not reveal differences in maternal satisfaction, but details regarding breastfeeding or maternal neonatal bonding were not studied. Except for the catheter therapy, the post-partum treatment protocol in the groups was similar.

Both the early catheter removal^2^ and early mobilization^20^ have been associated with a shorter hospital stay. The present study did not find any association with the time of catheter treatment, earlier mobilization, and the length of hospital stay. Since 76% (79/104) of our study population was discharged at, or before, the second post-operative day, the length of stay is already quite short. Furthermore, it is strongly associated with the length of newborn treatment and follow-up. Any actions to improve the patient care are unlikely to further reduce the length of stay.

### Strengths and limitations

The study setting can be considered a strength of this study. All patients were treated in the same hospital, by the same personnel, and following a standardized treatment protocol. Therefore, it is unlikely that the results are attributable to variations in treatment protocols. Additionally, the midwives’ documentation of mobilization was precise and reliable. Controlling for possible confounding variables related to post-operative mobilization is another strength of the study.

Our study has several limitations, primarily due to its retrospective nature. Parturients for the study were not randomized, as participation in the intervention group required voluntary consent for earlier-than-usual urinary catheter removal. This may have introduced selection bias, with more mobilization-capable parturients likely opting to participate. However, the actual time of catheter removal was most strongly associated with time to mobilization, not the participation in the study itself. In contrast, controls were selected by choosing the next Finnish-speaking parturient undergoing elective cesarean sections and not participating in the study. No attempts were made to control selection bias and standardize participants based on background characteristics. This approach was, however, considered appropriate given the relatively strict inclusion criteria when selecting parturients from a low-risk delivery unit. The parturients in this low-risk unit represent a relatively homogeneous group excluding morbidly obese, severely pre-eclamptic, and other parturients who may have greater difficulty mobilizing. This unit-specific selection led to a balanced distribution of cofactors between the intervention and control groups.

Another limitation is the lack of systematic postoperative pain assessment. However, upon discharge parturients must not need opioid medication, as these are not prescribed for home use in after cesarean sections. We did not study the patient’s activity after the initial upright mobilization because we regarded the first mobilization as the most critical event of mobilization. Consequently, some differences in and between the groups may have gone unnoticed. The low participation rate (16%) can be considered another weakness of this study. Since only native Finnish-speaking patients were recruited, 30% of the patients were excluded from recruitment. The high incidence of fear of childbirth may be associated with general fearfulness and uncertainty and a decreased willingness to participate in a study concerning new treatment protocols.

## CONCLUSIONS

The time to post-operative mobilization is associated with the duration of the catheterization. Since early removal of the urinary catheter and early mobilization are both key elements of the ERAS-protocol, the early removal of the catheter, may not only promote earlier mobilization but also motivate the parturient and staff in the recovery process. The relationship between the urinary catheter use and mobilization should be further studied in a prospective randomized trial including also the parturient perspective and experience on the optimal duration of the postoperative catheter treatment.

## Data Availability

The data supporting this research are available from the authors on reasonable request.
